# Active pharmaceutical ingredient (API) chemicals: a critical review of current biotechnological approaches

**DOI:** 10.1080/21655979.2022.2031412

**Published:** 2022-02-08

**Authors:** Vinod Kumar, Vasudha Bansal, Aravind Madhavan, Manoj Kumar, Raveendran Sindhu, Mukesh Kumar Awasthi, Parameswaran Binod, Saurabh Saran

**Affiliations:** a Fermentation Technology and Microbial Biotechnology Division, Csir- Indian Institute of Integrative Medicine (Csir-iiim), J & K, India; b Academy of Scientific and Innovative Research (Acsir), Ghaziabad-India; c Department of Foods and Nutrition, Government Home Science College, Affiliated to Panjab University, Chandigarh, India; d Division of Infectious Disease Biology, Rajiv Gandhi Centre for Biotechnology, - Trivandrum- India; e Deapartment of Food Technology, Tkm Institute of Technology, Kollam-India; f Department of Resource and Environmental Science, College of Natural Resources and Environment, Northwest A&f University, Shaanxi Province, Yangling, PR China; g Microbial Processes and Technology Division, CSIR-National Institute for Interdisciplinary, Science and Technology (Csir-niist), Trivandrum- India

**Keywords:** Pharmaceutical ingredients, biotechnological approaches, challenges, future prospects

## Abstract

The aim of this article was to generate a framework of bio-based economy by an effective utilization of biomass from the perspectives of agriculture for developing potential end bio-based products (e.g. pharmaceuticals, active pharmaceutical ingredients). Our discussion is also extended to the conservatory ways of bioenergy along with development of bio-based products and biofuels. This review article further showcased the fundamental principles for producing these by-products. Thereby, the necessity of creating these products is to be efficaciously utilization by small-scale farmers that can aid the local needs for bio-based materials and energy. Concurrently, the building up of small markets will open up the avenues and linkages for bigger markets. In nutshell, the aim of the review is to explore the pathway of the biotechnological approaches so that less chosen producers and underdeveloped areas can be allied so that pressure on the systems of biomass production can be relaxed.

## Graphical Abstract

**Figure:**
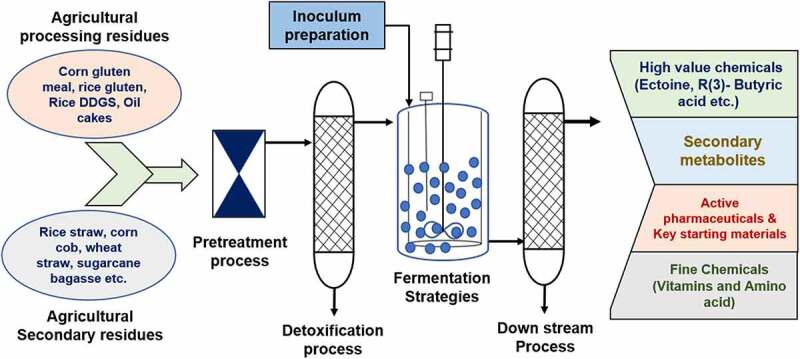

## 1. Introduction

Active pharmaceutical ingredients (APIs) are the chemical-based compounds that have produced mainly in the countries the USA, Europe, China, and India. APIs have pharmacological activity mainly used with combination of other ingredients to diagnose, cure, mitigate, and treat the disease. However, in the recent past years, many medicinal-based corporations have started importing these substances from countries producing active ingredients to their home countries. Modern day medicines have been used by people to prevent, treat, diagnose, and cure disease. Every single medication is composed of two main components, i.e. the API, which is the major component, is chemically and biologically active that has to do the work in your body and other component known as excipients like lactose or mineral oil in the pill, which is chemically inactive that provides, e.g. volume, a sweet flavor, or a color. These excipients helps in the delivery of APIs in the body system. Numerous chemical compounds and raw materials are utilized in multi-step reaction to make an API. (1–4) However, their main purpose is to treat the disease directly by acting upon (via their pharmacological activity) along with combination of inactive form. Nearly more than 1 lakh tones of pharmaceutical products are consumed all over the globe (e.g. Europe alone covers up to 24% of the consumption of medicinal products). Therefore, the generation of these APIs has stimulated the release of chemicals in the environment and thereby lead to the spreading of pollution. Concurrently, this enormous generation of pollution has called the worldwide attention that requires an immediate alteration in the policies and regulations [1,2].

Thereby, considering the negative impacts of these chemical channels that are responsible for generating API, the pharmaceutical companies are rendering approval to the microbial-based fermentation using bacteria or yeast [3,4].

Likewise, the inclination toward microbial-based manufacturing of recombinants has been increased in the past decade and these manufacturings are liable for the approvals of FDA for the human use. Adding to the fact, the microbial-based biopharmaceuticals created the returns of around $100 billion in 2017, and the business is escalating at a substantial pace (6% CAGR) [5,6]. It is predicted that in the year 2020 the market demand for fermentation-based protein drugs is expected to reach $60 billion from $44 billion. Similarly, the demand of peptide hormones and vaccine, which was $10–19 billion, respectively, is expected to reach $18–28 billion [7,8]. Today pharmaceutical companies such as Bayer, AbbVie, Biocon, GlaxoSmithKline, Eli Lilly, Sanofi, and Merck are depending upon microbial schemes and arrangements for the production of biopharmaceutical products. In this review, the focused has been laid upon the current and modern developments that taken place in chemical route of processing of biomass relative to traditional methods with emphasis on the production of APIs.

These APIs can be natural or synthetic chemical-based active compounds that are usually found in therapeutic and veterinary drugs. There are chemical-based active compounds produced using unsafe chemical routes. Therefore, their wide production, usage, and disposal are getting hazardous to human, water bodies (including drinking water), and other bio-based lives owing to their uncontrolled contact to the environment [3–5,7,9]. Furthermore, the presence of these compounds has been detected in the trace levels from nanograms to micrograms in the last span of 10 years from ground water, drinking water, and waste water [4,6–9]. The recent finding was reported on the pervasiveness of enormous amount of around 18 APIs in the Lake Victoria-Uganda (in the amount of 5600 ng L^−1^) [10]. Thereby, these APIs have been established as global contaminants. Although the formation of these chemical compounds is not from the single source of reaction, rather, they are developed from many chemical components that are usually initiated from a single intermediate [11]. Furthermore, several intermediates are formed during the process in order to convert any raw material into an API. These several reactions usually pass through the long channels of purification during their developmental engineering, which involves the usage of huge reactors [12]. Concurrently, these APIs are then checked for their purity before they are being sold to the drug manufacturers.

In order to generate the chemicals, one of the major biomasses is the carbohydrates, which constitute about the largest (95%) amongst the organic compounds of the planet. These are basically exploited for producing further products either using fermentation or chemical alteration. Biomass in the form of starch, cellulose, hemicellulose, pectin, and lignin usually exists in the form of feedstock [13]. In the earlier era of traditional processing starch and other carbohydrate-rich feedstock were usually being exploited as a raw material by the vivid chemical industries. Undoubtedly, various products in the form of chemical and polymers can be generated by using any alteration in the procedure (fermentation) in order to achieve the desired derivative.

Although there is a disquiet apprehension on the usage of starch between the food-based and chemical industries, that is why the application of this cellulosic biomass (lignocellulosic) has more inclination toward production of fuel, which is more than the conversion to xylose and glucose [14–16]. Since the earth’s biomass is largely composed of lignocellulose and thus the entire crop left over in terms of wheat and rice straw, sugarcane bagasse and fibers of corn husk are classified underneath of it [4]. These crops are made of lignin, hemicellulose, and cellulose, which are collectively called as lignocellulosic material. These lignocellulosic materials are processed by various techniques, viz. chemical, thermal, or biological; they are converted into sugars, chemicals, or ethanol [17–19].

But in order to breakdown these lignocellulosic materials, some pretreatment in the form of hydrolysis, delignification like the usage of sulfuric acid, alkaline treatment, high-temperature steaming, and pressure-based homogenization are generally required for distorting the structured and organized plants-based structure [20–25]. Therefore, after the application of pretreatments of these lignocellulosic materials, they can be readily converted to desired intermediates. In order to reduce the steps of production or to reduce the cost by keeping the safety of the environment, we need to cross the various technological and economic barriers for employing the potential applications of lignocellulosic materials [9].

A number of multiple published reports focus on the production of ethanol rather than other product [26,27]. Thereby, there are limited number of life cycle assessments (LCAs) that can generate multiple products. However, from the perspective of environments as well as economic, most of the bio-based fuels and bioresources that are produced under the one canopy of the factories are still not a promising option [28].

Similarly, forestry-based biorefinery system is one of the specific examples [29]. Concurrently, there are a number of key factors that have been recognized considering the environment-favored performance for the generation of bioethanol and biodiesel. In order to carry out the united production of chemicals, a widened and well-adapted spectrum is needed [30]. Nevertheless, there are still certain gaps of reasonable prices that are required for the creation of large-scale biorefineries. Furthermore, the chemical properties of biomass are not always favorable to biomass-based raw material; therefore, new technologies are demanding and challenging amongst the existing companies [31].

Also, propionic acid and its related esters are produced in the amount of approximately 192,000 tons throughout the year and also have several applications in the chemical industry (e.g. for the production of thermoplastics, solvents in paints and resins as well) and in the usage of animal feeds [32]. Further, from the viewpoint of biorefinery its production can be elevated for the fermentation of glycerol and sugar [33]. Also, economically, the conversion of glucose to propionic acid is anticipated to be 15% less than the conversion of glycerol. Likewise, the other related upcoming feasibility is to employ the fermentation of glycerol for the production of 3-hydroxypropionic acid, which is an essential building-block chemical [34]. Secondly, it can be converted to 3-hydroxypropionic acid to acrylic acid, which comparatively has better environment-based functioning. Concurrently, researchers from the National Renewable Energy Laboratory have reported the development of an innovative pretreatment by employing the use of an organic solvent in combination with water for the clarification of lignin and sugars derived from chemical nature [25,35].

Moreover, the contemporary revolution in the advanced technologies (e.g. genetic and metabolic engineering, enzymatic engineering) has opened up new avenues for creating vivid types of industry-based products such as APIs from raw materials derived from plant [4,6]. Owing to the preferential use of renewable raw materials of biomass by the APIs’ chemical industries, the concept of biorefinery has emerged and can be executed to produce different bioproducts with the replacement of chemical-based manufacturing [8,10]. Renewable resources are good sources as substrate for the production of green chemicals, APIs, and key starting materials (KSMs), which come under a vast subject. Therefore, the presented review entirely covers the major developments and possibilities of lignocellulose as feed stock that occurred in the recent years in the capacities of renewable biomass as a prominent foundation of chemicals and their related converted products.

## 2. Environmental issues associated with the production of API chemicals through chemical route

Presently, owing to the considerable increase in the pollution threat to the environment, more and more API-producing companies have been urged to follow the greener path in order to reduce the generation of waste (in terms of chemicals, solvents as by-products) [36,37]. Whereas API-producing companies are always looking for the faster and economical methods, however, in reality, if we tend to bring down the generation of waste, then the number of steps for producing API has to be reduced [38]. Because few and less tangible steps is the primary requisite with production of solvents or chemicals generated for producing a single pure molecule of API. Likewise, in addition to cut down the steps, manufactures are further needed to select nonhazardous types of solvents that possess the ability of producing proficient and effectual results [39,40]. Thereby, in order to achieve the results on greener guidelines, manufacturing companies should employ contract development officers (CMOs) and further pass their product through the services of contract development and manufacturing organization (DCMOs) so that the formulation of the process of API can be planned out at an early stage with the help of additional screening to avoid unavoidable changes or alteration in the later stage. This can be accomplished by following the protocols of scale-up processing in the pilot plants where thorough supervision needs to be administered for assessing time-to-time assessment and quality control. However, the establishment of such a kilo lab comes with potential challenges of good manufacturing practices (GMPs) that require planned budget and diligent supervision. And, by following these measures, we can reduce the exploitation of raw materials and generation of by-products [41,42].

Concurrently, in the last year’s vivid methods of organic synthesis have been employed for generating pharmaceutical products, which has strengthened the medical sector by reducing the causalities, illnesses, and death. However, in order to achieve this accomplishment, if we are deteriorating the environment simultaneously then all the efforts of pharmaceutical chemists will go in vain. Therefore, the pathway of green chemistry is utmost desirable for minimizing the dreadful impact on the environment. It is widely known that 80% of the wastage in the form of by-product by the pharmaceutical industry is related to the solvent reported by GlaxoSmithKline [43]. Thus, the production of the significant amount of the contaminated solvents will generate the air and other pollutants.

These ways can only be achieved through employing the use of sustainable tools. These tools call for the nib-to-nib strategy-based research that involves the following steps in making the processes threat free. The ways of biomass-generated feedstocks, their bioconversion routes, use of selected harmless chemicals, and channelized ways of technical processing are the key parameters that can lessen the environmental issues to the minimal. Similarly, the inline concept of integrated biorefinery and the continuous use of biosubstrates with the consumption of nonrenewable sources can safeguard the environment with a profound relevance [44]. In order to satisfy these parameters, the methodology of LCA needs to be persuaded that consisted of complete assessment of the products and processes from start to the end and quantify each environment-based quantification. Therefore, the only way to drop down the expanding problem is the generation of green manufacturing practices from pharmaceutical industries with thorough attentiveness on the selection, use, recovery, and disposal of the chemicals.

## 3. Method of biomasses conversion in APIs synthesis

Presently, the environmental amicably processes are becoming tremendously popular for the conversion of biomass to APIs. As following this route, we can be able to bring reduction in the rate of global warming. There are multiple processes that can convert lignocellulosic biomass to APIs in terms of fermentation to form pharmaceutical ingredients.

### 3.1. Chemical approach

The processes that transform the biomass to value-added chemicals (furfural, levulinic acid, etc.) in the presence of catalyst (hydrosulfuric, hydrochloric, and phosphoric acids) and conditions of high temperature and pressure fall under chemical conversions [38–40,44–46]. Although the factors of low yield are always being confronted as major challenges for commercialization. Therefore, in order to bridge the gap between the challenges, various innovative methods have been employed to convert biomass to chemicals. Likewise, a novel process was reported using catalysts based on modified carbon that were expanded to transform organic acids and sugar by the Northwest National Laboratory [44–48].

Furthermore, the performance of zeolites has been exploited for the conversion of biomass to APIs, and it was observed that the usage of zeolites was found to be remarkable owing to their selective size and shapes and they have been proved as potential catalysts [47,49]. In addition, the strength and constancy of silica-based catalysts were reported for converting glucose to sorbitol [46]. The research was carried not only in the field of innovated catalysts but also for the innovative pathways, and the routes were also investigated for the selective conversion of important chemical (e.g. production of hydroxymethyl furfural) [48,50]. Concurrently, the formation of acetic acid from biomass using supercritical water employing hydrothermal processing was also reported [49–53]. Moreover, there are certain chemical intermediates (3-hydroxypropionic acid) that also formed and foster the formation of final stage products as tetra-hydro furan (THF) and gamma-butyrolactone from 1, 4-diacids, and 1,3-propanediol [50–52].

There are certain monosaccharides like glucose and xylose that can be converted to different chemicals through the process of bioconversion following the route of chemical processing. Thus, glucose is an essential and foundational raw material for the bioindustry as crude oil is to the petrochemical industry. Sources of starch like corn, tapioca, wheat, and potato are profoundly known to produce glucose using enzymatic hydrolysis on an industrial scale [54]. According to the report by Kim and Dale [55], 3.5 million tons (MT) of lactic acid produced (10 times higher than the current annual lactic acid production) from using hydrolysis of approximately 1500 MT of crop residues and approximately 73.9 MT of fruits residues were obtained from rice, maize, barley, sorghum, and sugar cane.

### 3.2. Biotechnological approaches

The pathway of biotechnological approaches explored the usage of biocatalyst (enzymes) or cells for the transformation of biomass into utility chemicals. In nutshell, it is considered as one of the most easy, simple, and convenient methods for the formation of industrial products from biomass [4]. In contrast to chemical conversions that involve high temperatures and pressures, biological conversions are relatively mild. However, the concept of these biotechnological-based conversions is not the novel addition because earlier as well the various commercially used chemicals are being produced from yeast and bacteria (in terms of acetone-butanol, citric acid ethanol, lactic acid, etc.) [56,57]. The merits of less formation time of by-products and higher yield of product and selectivity (of biocatalysts) to convert renewable resources into chemicals have created fascination in recent time.

Although there are certain limitations in the processing of fermentation owing to various variations in the pathway of microorganisms, we cannot produce large variety of products [6]. Concurrently, there is a strong demand of novel processing techniques in order to widen the scale of products. Therefore, the only way to bring variety and resolve the limitations of biotechnological pathways is to involve the technologies based on genetic engineering and recombinant DNA technology, which can alter the gene coding and can bring about the desired changes for the sugar metabolism [58]. Likewise, modified *Escherichia coli* proved useful for the production of the compounds (catechol and adipic acid) from glucose. Recombinant *Saccharomyces* yeast converts glucose and xylose present in cellulosic biomass into ethanol [59].

Moreover, there is production of chemicals obtained from biomass using immobilized enzyme and whole cells. Huang and Yang [60] used rotating fibrous matrix of immobilized *Rhizopus oryzae* cells to produce fumaric acid from glucose and corn starch. Conversion of biomass hydrolyzate into chemical by absorbing it on solid metal oxide support using microbial process with compounds produced from lignin and fermentation inhibitors to enhance yield has been patented by Hames *et al*. [61].

That is why persistent efforts have been made for the alteration of enzymes and living organisms to produce the desired chemicals and particularly from the renewable sources. High yield and selectivity as well as minimal waste streams favor biological conversions as pathways for converting biomass into higher value chemicals. But there are numerous hindrances with the ongoing biological-based transformations (e.g. the higher energy requirements, lower production rates, continuous stirring) for achieving the desired results in bulk [3,6].

Biorefineries are largely required during the fermentation process of saccharides for their transformation to chemical-based by-products [62–64]. Thereby, this process fostered the raw materials to their complete degradation to unalloyed sugar solutions and generic feedstocks. Microorganisms are then used in fermentative medium to produce metabolic product in excess. Thereby, these metabolic products can further be utilized for the conversion of huge chemicals employing the biological or chemical route. However, the essential 12 molecules reported as the most favorable for the utilization in the conversion of biomass are arabinitol, aspartic acid, fumaric acid, malic acid, glutamic acid, glycerol, levulinic acid, malic acid, succinic acid, sorbitol 2,5- furandicarboxylic acid, 3-hydroxybutyrolactone, and 3-hydroxy-propionic acid [65,66]. A glucose-based media was proposed for the production of 3-hydroxypropionic acid and above mentioned chemicals using fermentation could be enhanced by using genetically engineered microorganisms and stated that from the chemical conversion of saccharides that obtained from biomass molecules namely glucaric acid, levulinic acid, 2,5-furandicarboxylic acid, 3- hydroxybutyrolactone [67,68]. Some important bioactive molecules that are produced from different biomass are summarized in Table 1 [69–80].

**Table 1.** Summary of chemicals produced from agro-biomass residues

**S. no.**	**Name of chemicals**	**Substrate name**	**Microbe’s name**	**Production process strategy**	**Yields**	**Reference**
**1.**	Diosgenin	Dioscorea zingiberensis	Mixed culture of *Trichoderma reesei* and *Aspergillus fumigatus*	Solid-state fermentation	95.82%	[69]
**2.**	Glutathione	Spent coffee grounds	*Millerozyma farinosa*	SmF		[70]
**3.**	2,5-furan dicarboxylic acid (FDCA)	Lignocellulosic biomass	Chemical route	Catalyzed synthesis	75%	[71]
**4.**	Glucaric acid	Corn stover	*Gluconobacter oxydans*	Two-stage fermentation	8.7 g/L/h	[72]
**5.**	Glutamic acid	Rice straw	*Bacillus subtilis* NX-2	Solid-stage fermentation	73.0 g /L	[73]
**6.**	Itaconic acid	Glucose	*Aspergillus terreus*	SmF	146 g/L	[74]
**7.**	1,2-Propendiol	Cellobiose	Beta-glucosidase-expressing *E. coli*	SmF	1.48 g/L	[75]
**8.**	2,4-Butanediol	Soy hull hydrolyzate	*K. pneumoniae*	SmF	21.9 g/L	[76]
**9.**	Isoprene	Poplar (*Populus* spp.)	Recombinant technology	-	-	[77]
**10.**	Lactic acid	beechwood	*Lactobacillus delbrueckii* sp. *bulgaricus*	SmF	51.6 g/L	[78]
**11.**	Ectoine	Rice straw hydrolyzate	*Halomonas elongata.*	SmF	377 mmol/kg FW	[79]
**12.**	R(3)-Butyric acid	Glucose, xylose, and arabinose	*E. coli PPA652ara*	SmF	1.38 g g^−1^ dry cell	[80]

### 3.3. Metabolic approach for API production

The branch of metabolic engineering is one of the empowering skill of science that has an eminent role in the expansion and progress of cell factories to further produce pharmaceuticals, fuels, chemicals, and food ingredients via following the route of microbial fermentations [69]. With the burgeoning and opening outgrowth of genetic engineering, it probably became much realistic to generate the miniature protein-based compounds like insulin, certain growth hormones employing the process of fermentation. Concurrently, metabolic engineering created the pathway for converting the minute microbes into cellular factories, owing to inexpensive raw materials like biomass-derived sugars to fuels and chemicals [81–84]. In the reported literature by Hong et al. [81], various industry uses of novel bioprocesses were shown for converting feedstock to agricultural-based products. One of its different biotech pathways was recently explored by DSM (Dutch multinational corporation) for the production of antibiotic cephalexin, which was earlier followed for the chemical conversion of penicillin. Similarly, Novozymes has formed a clubbed endeavor with Cargill with the interest of developing a bio-based procedure for the manufacturing of 3-hydroxypropionic acid. Nevertheless, Gevo has further created the opportunity of biofuel by following a process for the production of isobutanol. In addition, Amyris has further developed a yeast-based fermentation process for the production of farnese that can be employed as biodiesel and can also be converted into squalene, which has wide usage in cosmetics.

It was seen that CA is produced through the submerged strains of *Streptomyces clavuligerus*. Thereby, in order to increase the production, several wide strains have been explored by Ser et al. [85]. The most commonly available carbon source, i.e. glycerol, has also been employed in cultivating *S. clavuligerus* to produce CA, which was found to hike the cultivation up to fivefold [86–88]. Amongst the latest findings, the role of yeast *Saccharomyces cerevisiae* was also found as a major cell factory for producing vivid industrial products [81]. Similarly, our group is also working on this yeast for the production of butanol in order to use it further for the multiple applications in pharmaceutical industry and biofuels (e.g. biodiesel).

A multiple number of hypothetical studies have explained the utility of cell factory for producing a wide range of chemicals, viz., lactic acid, glycerol, and malic acid [85,89–91]. Moreover, the multiplicity of isoprenoids is further getting huge and thereby indicating the creation of other bioactive compounds through the medium of novel enzymes that can be explored for chimeric pathways [91]. For these reasons, there has been wide attentiveness in mounting microbial-related production of isoprenoids through following plant-based pathway with some significant genetic modifications of leader sequence and for relocating the relative genes [92].

## 4. Some important types of API chemicals

### 4.1. Shikimic acid

**Figure 1.**
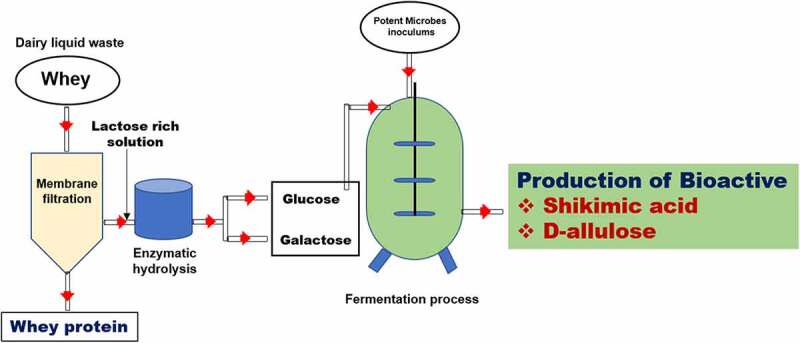
Systematic representation of utilization of dairy waste (whey) for the production of APIs.

In the pharmaceutical industry, shikimic acid is extensively used for the synthesis of chiral buildings block. Shikimic acid-based antiviral drug oseltamivir is used in the treatment as well as prophylaxis for the patients suffering from influenza (A, B). Shikimic acid also plays a key role in the development of an anti-influenza medicine termed as Tamiflu [93–97]. But the production of an increased amount of Tamiflu falls under the hazardous category. Owing to the existence of the conditions of mild reaction, careful handling is required as chemistry of potentially explosive is involved in the synthesis steps of this drug reaction. To bring down the uptake of shikimic acid by *E. coli* capable of synthesizing shikimic acid, methyl-α-D-glucopyranoside that mimic glucose was added to the culture medium as it is anticipated that transport systems of shikimic acid are controlled by catabolite repression [97]. Minimization of formation of quinic acid (0.09 to 0.01 mol/L) considerably throughout due to the incorporation of methyl-α-D-glucopyranoside as a substitute to glucose. The yield initially rose from 0.14 to 0.19 mol/L based on glucose and the yield of shikimic acid from 28 to 35 g/L due to the addition of methyl-D-glucopyranoside. Aromatic amino acids and aromatic vitamin can be assessed using shikimic acid with the help of strains of *E. coli* that blocked the first three steps of the Pittard and Wallace aromatic amino acid pathway [95]. Currently, the use of fermentative approaches on *E. coli* by Swiss pharmaceutical company named Roche produces shikimic acid [90]. However, the major concern is the production of large number of bioproducts produced during the synthesis of shikimic acid. It has been reported by various researchers that if we limit the carbon source during fermentation of shikimic acid large amount of bioproducts are formed. However, if the medium is replenished with high carbon source it leads to accumulation of high shikimic acid. It has been found that phosphate-limiting, carbon-rich growth conditions support shikimate production over by-products, and limiting carbon growth induces the formation of by-products [98]. This involves the blockage of aromatic amino acid pathway post shikimiate-3-phosphate (S3P) production. S3P was converted into shikimic acid owing to the activity of bacterial phosphatases. Draths *et al.* [99] found that in rationally engineered *E. coli* strains the production of shikimic acid by metabolic engineering is the most advanced after the amino acid pathway in these strains and the disruption of the K and L genes (that are responsible for encoding shikimate kinase I and II) was blocked. Furthermore, dairy effluent (whey) may be a good source of glucose extract from enzymatic hydrolysis of whey [100]. This glucose may be use as substrate for the production of shikimic acid (Figure 1)

### 4.2. Succinic acid

Succinic acid or butanedioic acid (C_4_H_6_O_4_) or amber acid is a common metabolite in plants, animals, and microorganisms and is further found in beer, coal, meat, eggs, honey peat, molasses, fruits, and urine. First purified from ‘amber’, the old fossilized resin of ancient trees, conifers in 1546, and has established wide applications till now. It has been known for curing alcohol hangovers by processing acetaldehyde, which is the most toxic metabolite of alcohol. Succinates are generally used for medicinal purposes as sedatives, antispasmers, antirheoters, contraceptives, inhibitor of potassium ions, and antioxidants [101–103].

The linear saturated structure of succinic acid has been acknowledged as the potential intermediate for the synthesis of industry-related chemicals of great relevance. Succinic acid has wide applications ranging from radiation dosimetry to agriculture, food, medicine, plastics, cosmetics, textiles, plating, photography, and waste gas scrubbing. It serves as feedstock for the manufacture of many commodity chemicals like polybutyrate succinate (PBS) and polyamides through esterification reactions [104,105]. Showa Highpolymer Co., Ltd., Tokyo, Japan, has been known to manufacture ‘Bionelle,’ a biodegradable plastic that is succinic acid and 1,4-butanediol ester that is the newest application of succinic acid [106].

**Figure 2.**
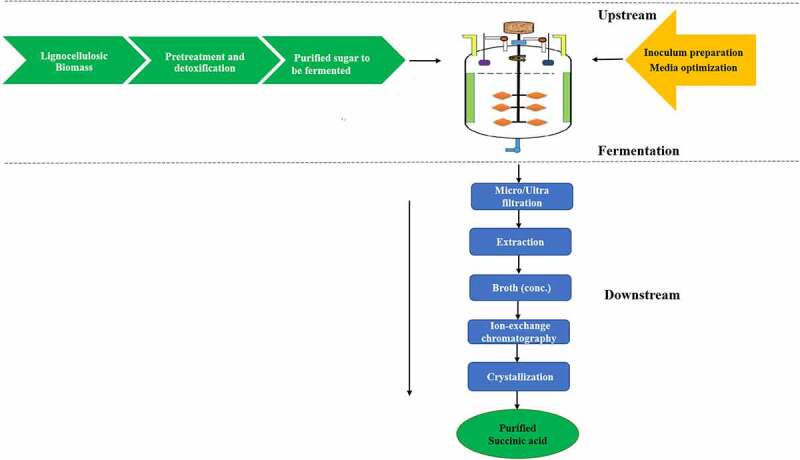
Systematic representation for the production of succinic acid using lignocelluosic biomass.

This is relatively due to the price of chemicals capable of forming succinic acid from maleic anhydride, which has greatly reduced its industrial applications [107]. Moreover, succinic acid is produced commercially through chemical synthesis by hydrolysis of petroleum products, lignocellulosic biomass (Figure 2), and also creates environmental issues [108]. Currently, green technology has become a driving force in the chemical industry in order to control pollution produced by processing of petrochemical and solves the issues for supply by basing production of hydrocarbons on a renewable environmental-friendly carbohydrates economy [108,109].

### 4.3. Erythritol

It is a sugar-based alcohol that has been regarded as a low-calorie sweetener all over the globe. Erythritol naturally occurs in various fruits and fermented foods [110]. It is usually generated from glucose with the process of fermentation (using *Moniliella pollinis*, yeast) and food-grade osmophilic yeast ferments the glucose that can be used for the production of erythritol [111]. It is nearly (of about 60–70%) a non-caloric sugar that at the same time neither alters the levels of blood suga, nor causes tooth decay. Food-grade osmophilic yeast ferments the glucose that can be used for the production of erythritol. Erythritol is purified after removed from the fermentative broth, and 99% purity was found in the final crystalline product. It does not pose any side effect of gastric-related problems owing to its distinctive metabolism of digestion [112]. Due to the occurrence of erythritol in foods, the US per capita, the consumption of erythritol is estimated to be 80 mg/person/day. It has been confirmed that body fluids of humans and animals contain erythritol. It has levels of approximately 1.2 mg/L in human plasma as well as fetal blood of animals. Human urine has 10–100 mg/L concentrations of erythritol [113]. However, renowned countries as Japan and the United States have already marked it as zero-calorie sugar, wherea, European regulations currently label it under nearly low calorie sugar (~0.24 kcal/g).

### 4.4. Clavulanic acid

**Figure 3.**
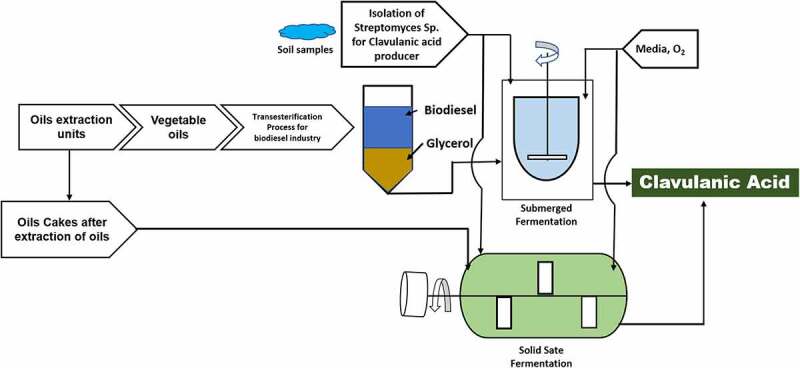
Utilization of processing residues (oil cakes and glycerol) from oil extraction and biodiesel production industry for clavulanic acid production through fermentation approach.

Clavulanic acid is one of the major medical drugs that are also affordable. It is employed to fight the bacterial resistance against β-lactam antibiotics and further it is included under the essential medicinal list of the World Health Organization. Taran *et al*. [114] worked on olive pomace oil (OPO), which is an industrial waste product from olive oil and has been explored as a substituted carbon source for the production of clavulanic acid by *S. clavuligerus*. Olive pomace oil was found to be six times more cost friendly than glycerol. It was estimated that OPO can be used as substitute for carbon source for clavulanic acid production. However, it was observed that OPO can further be employed as the potential carbon source (Figure 3). Likewise, Efthimiou *et al*. [115] reported olive oil formed from *S. clavuligerus* as the distinctive carbon and energy source for assisting clavulanic acid production. Results showed that production and yields are equivalent to those described by complex medium having oil. The variation in the recovered yields showcased the idea of different metabolic pathways for transforming the substrate into the desired product. Glycerol coupled with nitrogen source, namely, arginine, is the common carbon source used for the production of clavulanic acid using *S. clavuligerus* [116–118]. Carbon containing complex culture medium has been used when oils, including olive oil, are being used for antibiotic production in bacteria [119,120].

### 4.5. Cephalosporin (7-aminocepahlosporanic acid) antibiotics

This are obtained from cephalosporin and are considered as efficacious key drugs for curing the infections and diseases caused due to bacteria. Heterocyclic moiety of the cephalosporins has presently provided a vast range of drugs. Cefdinir, cefotaxime, cefuroxime, and ceftriaxone are some of the β-lactam antibiotics cephalosporin-based drugs that have been used in the market [121]. Ceftobiprole and its derivatives are the most recently developed and effective in the treatment of methicillin-resistant *Staphylococcus aureus*-based severe infections. The synthesis of 7-aminocephalosporanic acid (7-ACA) can be started from cephalosporin C, which is the most suitable starting material that can be obtained in required amount at a reasonable cost through microbial fermentation. Furthermore, cephalosporin C is a straight precursor that results in the formation of 7-ACA through amide bond cleavage [122].

### 4.6. Rifampicin

Rifamycin, also named as ‘Wonder Drugs,’ is a wide variety-based antibiotic with elevated demand for humans in drug therapy. It opposes the growth of broad range of pathogens including bacteria, eukaryotes, and viruses [123,124]. In solid-state fermentation (SSF), use of mutated strain of *Amycolatopsis mediterranei* OVA5-E7 for the production of rifamycin SV (1310 mg/100 gds) was optimized using cheap agro-industrial by-products using ragi bran [125]. The yield can be further escalated to 197 g/kg of dry substrate by an addition of deoiled cotton cake (10% w/w) into substrate with pH 7.0, 80% moisture content, incubation temperature of 30°C, 25% v/w inoculum percentage for 9 days under solid state fermenter conditions.

### 4.7. Pregabalin

Pregabalin is an efficacious anticonvulsant used for the treatment of seizure disorders, fibromyalgia, and neuropathic pain [126]. Stereo isomeric structure, (S)-enantiomer, is known for pregabalin pharmacological activity, so has its asymmetric synthesis acquired considerable importance in the pharmaceutical industry [127]. (S)-3-cyano-5-methylhexanoic acid, a key intermediate of (S)-pregabalin, has been asymmetric synthesized through biocatalytic and chemocatalytic routes Asymmetric synthesis of (S)-3-cyano-5-methylhexanoate, 98% and 97% of satisfactory enantiomeric yield from bisphosphine rhodium [128] and nitrilase [129], respectively, has been reported. Though both routes were unsuitable with respect to environment-related issues and economically as well [130].

### 4.8. Ectoine

When microorganisms grow in a medium with a high salt concentration, they produce primary metabolites and help adapt to an extreme environment. The primary metabolite is called ectoine [131]. Ecoine is a cyclic amino acid normally produced by halophilic microbes. Ectoine is a high-quality product and is widely used in cosmetics and pharmaceuticals. Ectoin can also be produced using lignocellulosic biomass and processing residues. Currently, ectoine is expensive to produce due to the higher substrate costs. Many researchers are also focusing on producing ectoine in an inexpensive manner by using a low substrate cost. Ectoin was extracted from fermentation broth using bacterial milking techniques produced by *Halomonaselongata* successfully 7.4 g/L achieved with 0.22 g/L/h productivity after at least nine cycles [132]. Production of ectoin on an industrial scale using the halophilic bacterium *H. elongata* (DSM 142T) in batch fermentation of 15% (w/v) NaCl followed by the separation of cells and ecoin extract from these cells by hypoosmotic shock was done [133].

## 5. Challenges and solutions to overcome the production of APIs chemicals through biotechnological route

This review emphasized numerous problems that are being confronted by big companies to industrialize the chemical and fuel synthesis technology (biomass collection and transport, biomass pretreatment, characterizations of treated biomass, fermentation, and chemical separation). Biomass location, terrain, type of residue, way into the field, and accessibility of machines are some of the aspects that influence the selection method for collection and harvesting of biomass. Biomass is collected using equipment and tractors with the help of labor. Baling choices include twine, plastic wrap, and net wrap. Source of biomass greatly determines the cost, e.g. biomass from forest residue and straw residue from cultivable land and small farms waste have different costs. After harvesting, distinctive methods are applied for biomass pretreatment. Supply of biomass to the industries plants can be either in trucks, ships, and trains based on the transport distance. Transportation of biomass using ships to a harbor is further unloaded and allocated by trucks for short distance and trains for long distances [134,135]. Large companies that have investment to introduce a new biomass-based biorefinery are currently irritating to bring different new (and lineup) process methodologies that still have numerous compound separation and compound purification challenges to resolve [136]. These include drying, chopping, grinding, shredding, pelletization, carbonization, and torrefaction. The process of torrefaction is a thermochemical process that comprises 50°C/min low heating rate, 200°C and 300°C of temperature range in the absence of oxygen. Another method widely used for decomposing biomass in the absence of oxygen is slow pyrolysis with temperature range between 400°C and 800°C and fast pyrolysis having temperature range between 450 and 550°C [137,138].

Characterization of biomass consists of particle size and shape characterization using mechanical sieving and laser diffraction. For flow and feeding properties of biomass angle of repose test (AoR), compressibility tests and shear cell tests are used. Other properties, namely, grindability, density, flowability, and energy content, have been estimated by hardgrove grindability Index, particle density, flow index, and oxygen bomb calorimeter method, respectively. Near-infrared (NIR) spectroscopy, which is a nondestructive approach, relies on specific bond detection and quantitation. Sequential extractive fractionation and hydrolysis that required very low sample mass can carry out comprehensive component analysis and nuclear magnetic resonance, which can be used for quantitation of sugars and biomass hydrolyzates, including both carbohydrate, hydrolyzate by-product compositions, and quantitation of fatty acids [139–142]. The method of biomass pretreatment and selection of lignocellulosic biomass should be finalized based on the accessibility of adequate amount of catalyst and substrate in that region. The lineup techniques are presently scaled up to launch pilot plants to validate the possibility. Concurrently, the logistics of biomass and techno-economic assessments are employed to evaluate the technology readiness level (TRL). Then assessments of the environmental impacts of the use of various technologies are created. Once suitable biomass feedstock, biomass pretreatment techniques, and enzymes are joined to synthesize inexpensive sugars, and then the selection of fuels and chemicals depends on the commercial market and can more significantly be derived from the biofuel strategy (implemented by the native and federal government). Additionally, in order to race with the price of fossil fuel, the price of biorefinery handling should be retained as less as possible by energy effective techniques and by the use of less amount of water. Synthesizing as numerous by-products as likely in a biorefinery will aid to decrease the price of fuel and chemical synthesis. If favorable situations persist later disabling these difficulties, then a large investment of about 100 million dollars is necessary to introduce an industrial biorefinery process that might synthesize several million gallons of alcohol per annum. It is significant that a biorefinery must be introduced in a suitable site that has enough water supply access to biomass substrates, and energy that is required for processing the substrates. Many large industrial corporations are placing all their capitals to first launch the lignocellulosic biorefinery and then remove all the obstacles to decrease the process price. Once this is finalized, then different technology groups will be sublicensed to various fuel and chemical manufacturers. The aim of launching a biorefinery concept is to synthesize energy in the condition of ethanol-based transportation fuel [143]. Consequently, it is highly significant to take the full advantage of the difference in energy expended and synthesized. Otherwise, the successfulness of a biorefinery concept will be assessed by the gross energy that is synthesized using the various processing steps [144]. Efficacies should be carefully checked to enhance the gross gain in the energy synthesized in the biorefinery [145,146]. In several techno-economic evaluations of second generation biorefineries, it is often reported that lignin will be a good source of energy for various processing steps. It is extensively presumed that biogas will be the major source of energy for running biorefineries, and as the techniques develops, the quantity of biogas utility will be gradually come down. Co-placement of biorefineries near coal or nuclear plants or utilizing energy from renewable sources is another strategy to receive power and heat [145]. Addition of an anerobic digestion plant nearby a biorefinery sector will be advantageous that could clear polluted water and at the same time the natural gas produced can be used for various biorefinery functionings [146].

In addition to energy, one of the major challenges that come across is the availability of the substrate [147]. The continuous supply of substrate is the primary prerequisite for keeping the uninterrupted production of the required ingredients. Further, the processes of recovery and purification (downstream processes) involved decide the destiny of the molecules formed from the biomass [148,149]. Moreover, the fate of fermentations costs, production costs, operation costs, etc., is one of the frontline challenges of this stream. Concurrently, the less tangible the downstream processes the more will be the yield. Therefore, the development of an economical method with an efficient downstream process is the indispensable requirement that poses the foremost challenge in the production of API. The overcoming of these bottlenecks is also the most technical requirement for potent generating pharmaceutical ingredients from the huge masses of biomass. Consequently, these challenges are highly significant for the processing of biomass wastes. Therefore, by converting the above challenges into strategies, the conversion of biomass to useful ingredients can be achieved efficaciously.

## 6. Future prospect of biomass to active pharmaceutical ingredient chemicals

To recycle the biomass and conversion of valuable bioproducts, streamline approaches are needed to continuous identify the novel technologies. However, utilization of biomass and conversion by the Active Pharmaceutical Ingredients Committee (APIC) in the circular bioeconomy noticed various challenges in terms of fulfilling the consumer demand and business opportunities. In addition, the exploitation of biomass and conversion of APC with circular bioeconomy has great advantages but still needs to put emphasis on proper handling of biomass and strict policy for waste recycling and management.

Recent biotechnology approaches like genetic engineering and bioengineering help to develop novel microbial strain or its consortium as well as advance model that mainly focused on improved various aspects like enhanced production of biomass yield, CO_2_ utilization, lipid accumulation, and bioremediation ability. Thus, integration of various technologies and computer engineering approaches will play a vital role on the way forward to achieve circular bioeconomy biomass-based valuable bioproducts like APC and biofuels. However, looking at present scenarios more questions have been raised than answers for the efficient utilization of biomass because it results in huge quantity of biomass like agricultural waste and it was burned either due to lack of knowledge or availability modern equipment. In addition, due to overexploitation of nonrenewable energy resources along with its high-cost chemicals it has forced the industries to identify sustainable, cost-effective, and renewal energy resources to look for long-term solutions for recycling of organic biomass. Thus, recycling of biomass and its conversion to APC is a significant approach for utilization of organic waste and biotransformation of valuable end products. Because in recent era, biomass recycling and conversion in APC has attracted a huge demand; however, still there are many critical problems and challenges associated with this method that are necessary to be identified properly and fixed by scientists worldwide. Looking all these aspects, integration of metagenomics techniques would be the valuable evidence in order to provide a better understanding of the mechanism of this process and metabolic pathways of microorganisms, its behavior to survive in their physical environment, and their approaches of system biology (such as proteomics, transcriptomics, and metabolomics).

Although several approaches are used to learn biomass to resource and its conversion in APC, a critical overview of the technologies used till date is limited and is not abundantly available. Hence, extensive research with molecular and biochemical tools is required to elucidate the remediation mechanism and focus on cost-effective and sustainable commercial exploitations. In addition, the combination of omics resources facilitates the production of more metabolites and bioactive compounds of interest that will ultimately lead to accelerating the drug discovery.

## 7. Conclusion

A huge quantity of biomass is getting collected either in the form of agricultural process or food processing/manufacture. Instant disposing of them without any treatment creates environmental threat, thereby more sustainable technologies are an attractive option to efficiently recycle it because its available in low cost and can be converted to multiproducts. In this review article, various economically viable technologies have been discussed comprehensively for biochemical conversion of agriculture biomass into valuable APC such as pharmaceuticals, biopolymers, bio-solar cells, fine chemicals, lubricants, etc. Advances in the technologies can generate bio-based economy and at the same time reduce the associated environmental risk. In addition, more future studies are the mandatory prerequisites in order to combine various technologies and restrict the secondary/tertiary pollution level to an efficient way through the channelized utilization of bioresources and to result in profitable business.

## Highlights

- Effective utilization of agricultural biomass for developing pharmaceuticals and APIs.
- Enhancement of the biobased circular economy.
- Economically viable technologies for biochemical conversion of biomass to API.
- Metabolic engineering is a promising tool for the production of API using biomass.
